# Prognostic Value of C-Reactive Protein in Primary Total Hip Arthroplasty

**DOI:** 10.3390/antibiotics14020205

**Published:** 2025-02-16

**Authors:** Moritz Mederake, Ulf Krister Hofmann, Georgios Eleftherakis

**Affiliations:** 1 Department of Trauma and Reconstructive Surgery, Berufsgenossenschaftliche Unfallklinik, University of Tübingen, 72076 Tübingen, Germany; 2 Department of Orthopaedic, Trauma, and Reconstructive Surgery, Division of Arthroplasty, Rheinisch-Westfälische Technische Hochschule University Hospital, 52074 Aachen, Germany; 3 Department of Orthopaedic Surgery, Diakonie-Klinikum Stuttgart, 70176 Stuttgart, Germany

**Keywords:** bone and joint infections, CRP, hip arthroplasty, infection parameters, orthopedic infections, periprosthetic joint infection

## Abstract

**Background/Objectives**: Periprosthetic joint infections (PJIs) are feared complications in arthroplasty and are associated with an increased mortality rate. PJI prevention is of paramount importance since treatment is difficult. In case of an infection, it is crucial to diagnose it at an early stage in order to initiate adequate therapy. The Musculoskeletal Infection Society (MSIS) proposed a catalog of different major and minor diagnostic criteria in 2011 to define a PJI. They were adapted in the following years. One of these criteria is the blood level of C-reactive protein (CRP). CRP is a non-specific acute-phase protein that also increases in response to various non-infectious inflammatory responses. CRP is also routinely obtained prior to total hip arthroplasty (THA) to screen for possible contraindications for arthroplasty such as an acute infection. The validity of this approach has rarely been investigated. The aim of this study was to evaluate the diagnostic value of perioperative CRP in patients receiving a THA. **Methods**: A total of 239 patients were included in this study and retrospectively analyzed. CRP values were obtained preoperatively and three values postoperatively. Sensitivity, specificity, area under the curve (AUC) and optimal thresholds were calculated. **Results**: In the whole group, 10 patients developed a PJI. No significance was demonstrated between patients without and with later PJI in terms of preoperative CRP (*p* = 0.182), postoperative CRP (*p* = 0.167), relative CRP increase (*p* = 0.684) and respective CRP differences (*p* = 0.456). We were not able to find cut-off values with adequate sensitivity and specificity. **Conclusions**: Perioperative CRP values do not seem to be helpful in predicting further PJI. Rather, they should be used as a screening tool to detect ongoing infections in the individual patient prior to THA. This trial should encourage studies with more statistical power due to the small effect sizes.

## 1. Introduction

Total hip arthroplasty (THA) results not only in significant pain relief but also in increased joint mobility for most patients [1,2]. Some challenges still remain despite the high level of clinical success which is achieved with modern arthroplasty techniques [3]. A feared complication is a periprosthetic joint infection (PJI), which is associated with increased mortality [4]. With an incidence of 0.2–2% for primary THA [5,6,7], PJIs represent one of the most frequent indications for revision surgery on artificial joints [8]. PJIs account for 15% of all revision surgeries after primary THA [9]. Besides the associated morbidity and mortality for the patient, PJIs cause a huge economic burden [10,11,12]. Prevention is thus of utmost importance. In case of a present PJI, it is crucial to diagnose it at an early stage in order to initiate an adequate therapy. In 2011, the Musculoskeletal Infection Society (MSIS) proposed a series of major and minor diagnostic criteria to define a PJI, which were adapted in the following years [13]. These criteria include results from clinical evaluation, microbiologic, histopathologic, joint aspirate and serologic tests [14]. In the latter, the erythrocyte sedimentation rate (ESR) and C-reactive protein (CRP) in particular play a decisive role. CRP is a non-specific acute-phase protein that also increases in response to various non-infectious inflammatory responses. It cannot identify localized contained infections [15]. Since it is cost-effective and simple to collect, it is still used by default to look for a present PJI [15,16,17].

Another setting in which CRP values are regularly evaluated is perioperatively to prevent PJIs. In this context, CRP is used as a screening tool for an ongoing infection which might increase the risk of early bacterial colonization of the newly implanted endoprosthesis. Especially in pneumonias or lower urinary tract infections without symptoms, CRP is able to initiate further diagnostics for the detection of these infections [18]. The current literature focuses primarily on revision surgeries when considering the relationship between elevated preoperative CRP and the incidence of PJI [19,20,21]. Evidence of the perioperative role of CRP values in primary arthroplasty is still scarce.

The present retrospective study analyzes the role of perioperative CRP and its association with the incidence of PJIs after primary THA. The purpose of the study was to answer the following questions:

1. Do cases with and without later PJI differ in terms of preoperative CRP values? How predictive is the preoperative CRP value?
2. Do cases with and without later PJI differ in terms of postoperative CRP levels? How predictive is the postoperative CRP value?
3. Do cases with and without later PJI differ in terms of the perioperative course of CRP (absolute and relative CRP increase)?
4. What is the optimal cut-off value in this context and how high is the sensitivity and specificity of this CRP value?

## 2. Results

A total of 239 patients were included in this study, having received a THA between 2009 and 2019. Patients who received a THA contralaterally during the course of the study were counted as new patients, and the two different sides were analyzed separately. Of the patients, 107 (44.7%) were female and 132 (55.2%) were male, with the average age being 79 years, ranging from 29 to 94. Of the implanted prostheses, 14.6% were cementless, 40.6% were hybrid (cemented stem and cementless cup) and 44.8% were cemented (Table 1).

The overall median follow-up period for the study population was 63 months with a range of 24 to 156 months. 10 patients (4%) developed PJI, which occurred within the first two postoperative years in all cases.

A total of 232 patients with chronic inflammatory diseases (e.g., Crohn’s disease, malignant neoplasms, rheumatoid arthritis, polymyalgia rheumatica) were excluded from this study. With respect to other comorbidities, 144 patients (60.3%) suffered from arterial hypertension, followed by 45 patients (18.9%) with coronary artery disease and 41 patients (17.1%) with type 2 diabetes mellitus (Table 2).

**Figure 1:**
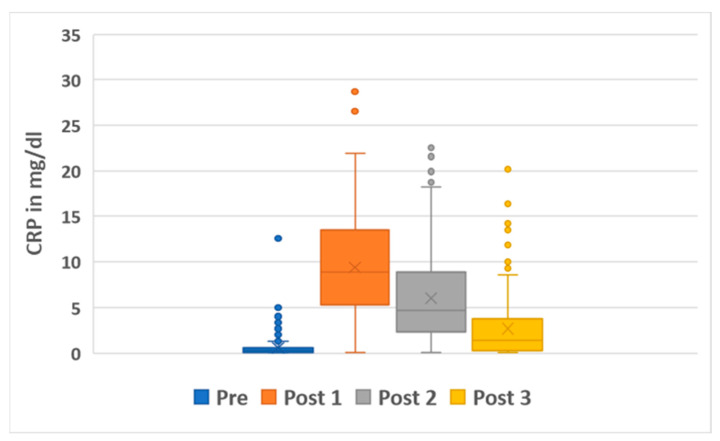
Boxplot diagram of C-reactive protein levels preoperatively (“Pre”), and the first (“Post 1”), second (“Post 2”) and third (“Post 3”) values postoperatively. Explanations: median: vertical line; mean: X.

The median preoperative CRP value of the total group was 0.26 mg/dL (0.01–12.58 mg/dL). The three postoperative median CRP values were 8.91 mg/dL (0.02–28.67 mg/dL), 4.73 mg/dL (0.01–22.53 mg/dL) and 1.4 mg/dL (0.01–20.21 mg/dL) (Figure 1).

Overall, a median increase of 8.5 mg/dL (−0.29–27.81 mg/dL) was observed when comparing the preoperative CRP level to the first postoperative CRP level. The CRP level increased significantly in the whole group due to the surgical intervention (*p* ≤ 0.001). Both in cases without (*p* ≤ 0.001) and with future infection progression (*p* = 0.005), CRP levels increased significantly from pre- to postoperatively.

Nevertheless, no significant difference was demonstrated between the two groups in terms of preoperative (*p* = 0.182) or postoperative CRP values (*p* = 0.167) and their relative (*p* = 0.684) or absolute increase (*p* = 0.456). Care must be taken when interpreting the data as due to the number of patients with later infection, the test power is only weak with a β-value between 0.92 and 0.95 (Table 3).

**Figure 2:**
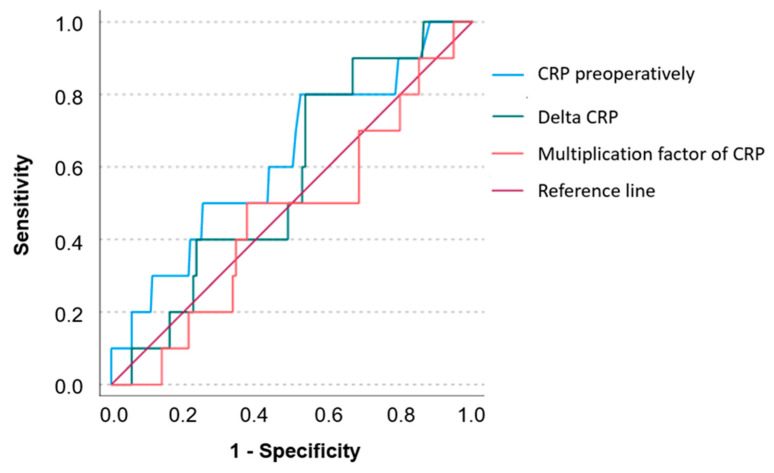
Area under the receiver operating characteristic curve for preoperative C-reactive protein (CRP) (“CRP preoperatively”), the respective absolute CRP difference (“Delta CRP”), and the relative CRP increase (“Multiplication factor of CRP”).

Furthermore, cut-off values were determined: For the preoperative CRP, the value was set at 0.235 mg/dL. The sensitivity was 0.8 and the specificity 0.476. The cut-off value for the CRP difference was 7.785 md/dL with a sensitivity of 0.8 and a specificity of 0.463. The cut-off value for the relative CRP increase was a factor of 44. The sensitivity in this case was 0.5 and the specificity 0.624. The area under the curve (AUC) data for each lab test to determine diagnostic effectiveness were further obtained. With an AUC of 0.625, 0.570 and 0.462 for preoperative CRP and the absolute and relative CRP increase, respectively, these parameters were shown to be only slightly more informative than chance (Figure 2).

Based on the effect size d and the PJI incidence of 4% in our cohort, we calculated the required sample size for an adequately powered study. The target power value was set at 0.8 (Table 4).

## 3. Discussion

There is currently no consensus in the literature as to whether preoperative CRP values can be used as a predictor for PJI after THA [22]. Furthermore, most available studies on this topic examine the relationship between perioperative CRP values after revision arthroplasty and the incidence of reinfection [23,24,25]. With regard to the question of how preoperative CRP values correlate with the rate of PJIs after primary arthroplasty, only a few studies can be found: Godoy et al. (2016) for example, were unable to show a statistically significant correlation between postoperative complications and preoperative CRP values (elevated CRP was classified as >8.2 mg/L) in a cohort study with 351 study participants who underwent unilateral primary total knee arthroplasty [22]. In comparison, Xu et al. (2018) demonstrated a significantly increased incidence of PJIs after the implantation of a primary TKA in subjects with preoperatively elevated ESR (elevated ESR was classified as >30 mm/h) and CRP (elevated CRP was classified as >10 mg/L) values by retrospectively analyzing 3376 patients [26].

The literature focusing on primary THA and the diagnostic value of perioperative CRP is scarce. Rohe et al. (2022) evaluated perioperative CRP in a retrospective case–control study on primary THA. In contrast to our study group, they focused on acute PJI and excluded late PJI. Taken together, perioperative CRP values failed with regard to diagnostic accuracy (sensitivity <80% and specificity <70%), and the authors question the usefulness of regular CRP evaluation postoperatively [27]. Inoue et al. (2024) analyzed 1115 patients undergoing THA in the first week postoperatively and divided them into three groups based on the preoperative CRP value. PJI was more likely in groups with higher CRP values; however, the incidence did not differ significantly between groups [28]. Another study analyzing inflammatory parameters and joint aspiration postoperatively was conducted by Dugdale et al. (2022). They focused on the first three months postoperatively and calculated optimal cut-off values. For CRP, they identified ≥100 mg/L within six weeks and >33 mg/L within six to twelve weeks postoperatively as cut-offs for suspecting PJI [29]. Furthermore, we were able to find two studies in the literature that had to do with both primary knee and hip joint arthroplasty. Pfitzner et al. (2008) retrospectively analyzed 50 patients who underwent primary knee or hip arthroplasties [30]; 25 patients with postoperative PJI were compared to a control group of 25 patients without PJI after primary arthroplasty. Even though preoperative CRP levels were higher than in the control group, no statistical significance could be found. Ghosh et al. (2009) conducted a prospective study with a cross-sectional design. Pre-, peri- and postoperative CRP values were collected from 121 patients who underwent total knee or hip arthroplasty, and they were divided into groups with normal (>3 mg/dL) and elevated (>3 mg/dL) preoperative CRP values [31]. A statistically significant relationship between a longer operative time and more complications could be found at the 14th postoperative day in patients with elevated preoperative CRP. The results in the systematic review by Domecky et al. (2023) resemble those described herein. There is no consensus regarding the diagnostic value of perioperative CRP. While there are studies presenting potential benefits, there are others questioning its diagnostic value [32].

Our incidence of PJIs after THA implantation of 4% is in line with other studies that have come to similar results [33,34,35,36]. Overall, a median increase of 8.5 mg/dL (−0.29–27.81 mg/dL) was observed when comparing the preoperative CRP level to the first postoperative CRP level. The CRP level increased significantly in the whole group due to the surgical intervention (*p* ≤ 0.001). Both in cases without (*p* ≤ 0.001) and with further infection (*p* = 0.005), CRP levels increased significantly from pre- to postoperatively. No significant difference was demonstrated between the two groups in terms of preoperative CRP (*p* = 0.182), postoperative CRP (*p* = 0.167), and relative (*p* = 0.684) and absolute CRP differences (*p* = 0.456). Therefore, perioperative CRP values seem not to be helpful in predicting further PJI. It must be taken into account that in our department, as well as in other arthroplasty departments, patients with relevant elevated CRP values were not operated on without further infection diagnostics [37]. This is reflected in the boxplot in Figure 1. With a few exceptions, all patients with elevated CRP (usually values > 2 mg/dL) values were subjected to an infectious disease focus search preoperatively, and any infection found was treated. This strict concept arises from the fact that in the case of active infections, significantly higher rates of PJI after total joint arthroplasty have been shown [38]. Due to this selection, which is not systematic but still exists, a bias results. Regarding this bias, the absolute and relative increases seem to be more reliable than the preoperative CRP values. This must be viewed as a limitation. However, performing surgery in patients with highly elevated infection parameters without further diagnostics and treatment is unethical and should not be carried out.

It must be kept in mind that most reasons for PJI are found intraoperatively or are related to postoperative ongoing infections [39]. Consequently, preoperative CRP is not predictive in these cases. However, although not statistically significant, the sensitivity of 0.8 for preoperative CRP and the absolute difference from the postoperative value should not be underestimated, since it can help in detecting ongoing infections which can lead to further PJI.

Regarding the major limitation of this study, our work is limited to 239 patients, of which 10 developed later PJI. Due to the small effect sizes, this leads to a significantly limited statistical power of the applied tests. The post hoc power analysis revealed that a type II error of non-significance cannot be excluded. Therefore, this trial suggests that perioperative CRP is not useful in predicting PJI; however, no definitive conclusions can be drawn from this study due to its underpowered size. Our study should encourage to conduct future trials on this topic with adequate power. To prevent further underpowered trials, we performed a calculation of the size of future samples based on our results.

## 4. Materials and Methods

### 4.1. Patients and Research Workflow

This study was conducted according to the guidelines of the Declaration of Helsinki and was approved by the local ethics board of the University Hospital of Tübingen (registration number 743/2024BO2). 

**Figure 3:**
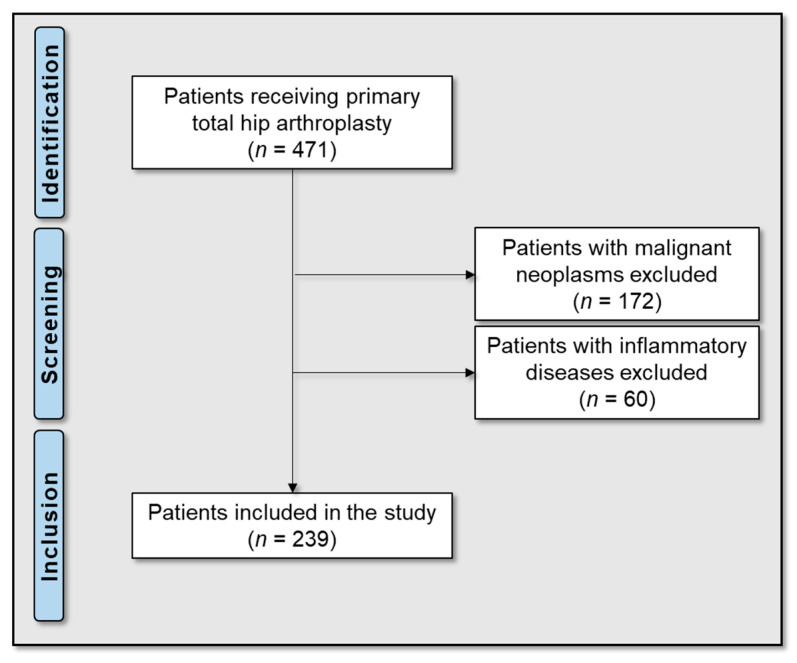
Flow diagram of the study group.

All patients who had received a THA between 2009 and 2019 in the Orthopaedic Department of the University Clinic Tübingen were reviewed. Of the 471 patients, 172 with a malignant neoplasm and 60 with an inflammatory disease (rheumatic disorders, chronic inflammatory bowel disease, vasculitis) were excluded (Figure 3). The medical records of all patients were reviewed for age, sex, body mass index (BMI), American Society of Anesthesiologists (ASA) score, prior hip surgeries, the type of implanted prosthesis, comorbidities, laboratory parameters, follow-up and outcome (later PJI or free from infection). The groups with and without later PJI were subdivided and then compared according to the predetermined objectives.

### 4.2. Treatment Protocol

Follow-up examinations took place at regular time intervals for at least two years. Classification as free of infection was made according to Diaz-Ledezma et al. [22] if the patient met the following criteria: free from mortality related to PJI, free from subsequent surgical intervention for PJI, and microbiological and clinical absence of the infection for at least 2 years. In case of suspicion of an infection at any time of follow-up, the MSIS criteria 2014 and the ICM criteria 2018 were applied. 

### 4.3. Laboratory Parameters

CRP (mg/L) was measured by a particle-enhanced turbidimetric immunoassay (Cobas C303; Roche, Basel, Switzerland) according to the manufacturer’s recommendations. The values were recorded at four defined time points: prior to THA, on the first postoperative day and two more values in the first week after THA. In-house thresholds—in order for a patient to be assessed as positive with regard to an infection—were set at ≥0.5 mg/dL for CRP. Changes were named “ΔCRP” and calculated using values prior to THA minus values postoperatively. 

### 4.4. Statistical Analyses

Statistical analyses were conducted using IBM SPSS Version 24 (IBM Corp., Armonk, NY, USA), G*Power (Version 3.1.9.7) and Microsoft Excel (Microsoft, Redmond, WA, USA). Distributions of variables within the groups were assessed by histograms and the Shapiro–Wilk test. Continuous variables are presented as medians (range) or means (standard deviation) as appropriate and categorical variables as frequencies. Comparisons were performed by the Mann–Whitney U test and Wilcoxon test as appropriate. All reported *p*-values are two-sided, with an alpha level of 0.05, and have not been adjusted for multiple testing. To determine the effect size of statistical tests, Cohen’s d was calculated.

Receiver operating characteristic (ROC) analyses and curves were performed and generated to determine the diagnostic value of the diagnostic tests regarding reinfection. The area under the ROC curve (AUC) was calculated as a measure of diagnostic effectiveness, and values were classified as follows: AUC < 0.6 fail, AUC 0.6–0.69 poor, AUC 0.7–0.79 fair, 0.8–0.89 good, and AUC 0.9–1 excellent [23]. To calculate the optimal threshold value of the laboratory tests, Youden’s J-statistics were applied. 

## 5. Conclusions

With our study group, we were unable to demonstrate a statistically significant association between preoperative CRP levels and the occurrence of PJIs after primary THA. It must be kept in mind that for all patients, the operation was highly elective, and this is why patients with severely high CRP values without further diagnostics and therapy were not assessed for surgery. In this regard, the absolute CRP differences and the relative CRP increase are more reliable. However, there was also no statistically significant difference. We were not able to find cut-off values with adequate specificity. Taken together, our findings demonstrate that perioperative CRP as well as the absolute CRP differences and the relative CRP increase are not helpful in predicting further PJI. CRP, rather, should be used as a screening tool for ongoing infections pre- and postoperatively to initiate adequate treatment to reduce the risk of hematogenous PJI.

The major limitation of our trial is the limited statistical power due to the small effect and sample sizes. Therefore, care must be taken when interpreting the results of our study. However, our findings help in estimating the sample size required for adequate power in future trials on this topic. In such studies, there will always be the ethical limitation that patients with severely high inflammation parameters cannot be assessed for surgery without adequate treatment. Alternatively, groups of patients with proximal femur fractures and THA treatment could be used, since surgery is usually undertaken in an emergency with less regard to inflammation parameters.

## Figures and Tables

**Table 1:** Implanted prostheses.

Type	*n*	%
Cementless	35	14.6
Hybrid	97	40.6
Cemented	107	44.8

**Table 2:** Pre-existing illnesses.

	*n*	%
Arterial hypertension	144	60
Coronary heart disease	45	19
Type 2 diabetes mellitus	41	17
Atrial fibrillation	25	10
Obesity	22	9
Hyperlipidemia	11	5
Cardiac infarction	11	5
Peripheral arterial disease	8	3
Renal insufficiency	7	3
Apoplexy	6	3
Chronic obstructive pulmonary disease	3	1
Pulmonary embolism	2	1
Deep vein thrombosis	1	1

**Table 3:** C-reactive protein (CRP) values for patients without and with later periprosthetic joint infection.

	CRP Preoperatively [mg/dL] (Median/Range)	CRP Postoperatively [mg/dL] (Median/Range)	Absolute ΔCRP [mg/dL] (Median/Range)	Multiplication Factor of CRP (Post-/Preoperative) (Median/Range)
Without infection	0.26 (0.01–5.5)	8.86 (0.02–28.67)	8.5 (−0.29–27.81)	29.38 (0.15–1369)
With infection	0.45 (0.05–12.58)	11.3 (2.85–19.46)	8.37 (2.28–18.07)	29.39 (1.47–157.4)
*p*-Value	0.182	0.167	0.456	0.684
Statistical power	0.08	0.08	0.06	0.05

**Table 4:** The required sample size of patients for a study with a target power of 0.8 based on the 4% incidence of periprosthetic joint infections after primary total hip arthroplasty.

	CRP Preoperatively	CRP Postoperatively	Absolute ΔCRP	Multiplication Factor of CRP
Effect size Cohen’s d	0.173	0.18	0.096	0.053
Sample size	1298	1198	4206	13,792

## Data Availability

Data are available from the authors on reasonable request.
